# Technical requirements framework of hospital information systems: design and evaluation

**DOI:** 10.1186/s12911-020-1076-5

**Published:** 2020-04-03

**Authors:** Mehrdad Farzandipour, Zahra Meidani, Ehsan Nabovati, Monireh Sadeqi Jabali, Razieh Dehghan Banadaki

**Affiliations:** 1 Research Centre for Health Information Management, Kashan University of Medical Sciences, Kashan, Iran; 2 Department of Health Information Management & Technology, Kashan University of Medical Sciences, Kashan, Iran; 3 Dr. Shari’ati Hospital, Hormozgan University of Medical Sciences, Hormozgan, Iran

**Keywords:** Technical requirements, Hospital information system, Communication service, System architecture, Security service, System response time

## Abstract

**Background:**

Implementing the health information system (HIS) is more complex and costly than implementing other information systems. The present study was conducted to design and evaluate technical requirements for the HIS.

**Methods:**

The present study was conducted in 2016 by determining technical requirements for the HIS using the Delphi technique and then evaluating this system using a checklist based on the approved requirements.

**Results:**

The first part of the study designed a 73-item final list of technical requirements for the HIS in four domains, i.e. communication service, system architecture, security service and system response time. The evaluation results obtained in the second part showed that communication service was met in 63.8% of the HIS programs, system architecture in 65.5%, security service in 72.4% and system response time in 76.3%.

**Conclusions:**

A technical evaluation tool was designed and used to select and evaluate the HIS. The evaluation results suggested the study HIS was poorer in terms of communication service and system architecture than in the other two dimensions.

## Background

Modern advances in information technology (IT) have completely transformed the face of the world, and IT-based services have significantly improved healthcare services [1, 2]. Among those, HISs are one of the most *commonly used* health information systems [3]. Despite the potential benefits, HISs are neither widely used by healthcare providers nor are they readily accepted by users where they are launched [4]. Given the significant failure rate of the HIS, efforts are required to be made to enhance its currently-slow popularity by meeting the implementation challenges of this system [5].

Failing to properly identify technical requirements for the HIS and its integrity are implementation challenges of this system [6]. Designing a framework for determining the technical requirements and accordingly evaluating the system is therefore crucial. Technical dimensions of information systems such as architecture, communication service and response time play a key role in the failure rates of these systems [7]. The technical factors have been addressed as the second leading or even the main obstacle to effectively implementing the HIS [1, 8]. Meeting technical requirements ensures information security and confidentiality as well as the successful communication of the system with other systems [9]. Determining the technical requirements assists network designers in identifying technical features and their supporting software version and also in selecting hardware platforms [10].

Given the significant relationships between technical factors and success rates of the HIS [11], establishing appropriate evaluative criteria is as crucial for overcoming technical obstacles to the system implementation [12] as accurately assessing the system is essential for its successful implementation [13].

The HIS evaluation and technical requirements have been addressed in literature in two domains or in a general manner in a single setting using a small sample and a maximum of 7 items [14–18]. Moreover, hardware, software, networking and technical support have been mentioned as the technical challenges facing the HIS development [19, 20]. The Iranian Ministry of Health and Medical Education has defined technical requirements for the HIS in two domains of communication services and security services [21]. A review of literature suggests the very limited number of requirements defined using a small sample mostly involve a small section of hospitals. Library research was also found to have failed to determine comprehensive requirements. The only detailed list of requirements defined by the Iranian Ministry of Health and Medical Education can be developed in terms of the defined domains and items.

Regarding the program of Iranian Ministry of Health and Medical Education for the application of IT services in the healthcare sector and development of electronic health records (EHRs) [22] and considering HISs as good examples of EHRs [23], implementation principles and the technical requirements of these systems need to be continuously assessed and revised [24]. The present study therefore proposed a comprehensive framework for defining technical requirements for the HIS and evaluating this system accordingly. These requirements are recommended to be employed by hospital executive teams and HIS developers.

## Methods

The present study was conducted in two parts, i.e. designing technical requirements for the HIS and its evaluation based on these requirements.

### First part: designing technical HIS requirements

This part determined technical requirements for the HIS in three stages: Focus Group Discussion (FGD), Modified Delphi Technique and Classical Delphi Technique.

#### Focus group discussion

Ten experts with at least a master’s degree in software, networking or IT and 7 years of work experience in IT departments of hospitals or medical science universities were first selected from faculty members and the staff. Before holding FGD, a panel of experts was presented with a primary checklist extracted from the comprehensive outline published by the Iranian Ministry of Health and Medical Education regarding HIS assessment criteria [21].

The challenges and technical obstacles to implementing the HIS were discussed in four 4-h sessions. The FGD was held to identify the major technical requirements from expert perspectives and determine all the technical and implementation problems that had ever faced the experts. At this stage, the experts freely exchanged ideas and discussed technical requirements for the HIS through brainstorming. The expert comments were recorded and then transcribed during the meetings. A list of technical requirements was ultimately developed and they were classified into four domains, i.e. communication service, system architecture, security service and system response time according to the expert comments. The primary questionnaire was then designed as the framework of technical requirements for the HIS, which included the study introduction and objectives and a list of technical requirements for the HIS with an open-ended question for including future expert comments.

#### Modified Delphi technique

The Modified Delphi Technique was employed at this stage to reach consensus on the requirements by going through a multi-stage process. As an open-ended round Delphi guided by focus groups or one-to-one interviews, this technique is generally used as a Group Delphi Technique to reach consensus using expert comments and structured questionnaires [25]. Experts present at this stage were those who participated in the FGD. At the end of the focus group, the initial technical requirements for the HIS were individually scored by the experts on a five-point Likert scale ranging from 0:strongly disagree to 4:strongly agree. The requirements receiving a total mean score of over 3 were approved, those with a total score of below 2 were eliminated and those with a mean score of 2–3 were reassessed by the experts to be either approved or removed from the questionnaire. The experts were encouraged to freely express their ideas and propose potential technical requirements for the HIS. They unanimously finalized the HIS technical questionnaire after holding four focus group rounds. Four groups of technical requirements for the HIS were ultimately accepted as a 72-item questionnaire with a mean score of 3.4.

#### Classical Delphi technique

At this stage, the 72-item questionnaire approved in the Modified Delphi Technique was distributed among 40 IT experts and IT managers of hospitals and medical science universities across the country with at least a master’s degree and a minimum of 5 years of work experience in the HIS field. This questionnaire included demographic information, 72 closed-ended items and an open-ended question to explore the possibility of including additional technical requirements (Additional file 1). The respondents scored the items on a five-point Likert scale ranging from 0: strongly disagree to 4: strongly agree. At all rounds of the Delphi technique, the participants were anonymously provided with feedback. To meet all the Delphi requirements, including anonymity and feedback, research collaborators were introduced to the study hospitals as another focal point. They were in charge of briefing the IT experts and managers on the study objectives and providing them with feedback on the data obtained at individual rounds. Thirty eight of the 40 questionnaires distributed were completed. The mean scores of the individual requirements calculated in SPSS Statistics for Windows, version 18.0 (SPSS Inc., Chicago, Ill., USA) were employed in statistical analyses. The Classical Delphi Technique was performed in two rounds. The ultimately-approved 73-item questionnaire included one additional item and four domains of technical requirements for the HIS with a mean score of 3.6.

### Second part: evaluating HISs

After inquiring of the Iranian Ministry of Health and Medical Education about the number of companies supplying the HIS, the software was found to be provided by 19 software suppliers throughout the country, 16 of which consented to have their HIS evaluated and provided a list of hospitals in which their system was being run for at least 5 years. A total of 16 hospitals, each corresponding to one of the suppliers, were then randomly selected to evaluate the HIS program. Running all the software modules was the only inclusion criterion for selecting a hospital. After completing the HIS technical checklists containing Yes/No items designed in the first part through individually asking from the IT users and experts working at the IT units of the selected hospitals as well as observing the implemented system, the data collected were analyzed in SPSS-18. The hospital HIS received a score of 1 if it met the checklist criteria; otherwise, it received a score of 0. The system performance was evaluated in individual domains as a score in percentage, with 0–20% denoting a very poor performance, 20–40% a poor performance, 40–60% an average performance, 60–80% a good performance and 80–100% a very good performance.

### Ethical considerations

Before beginning the FGD, all the participants signed informed consent forms, in which they were briefed on the study objectives and their voluntary participation in the FGD and confidentiality of their information were ensured. Before completing the checklist in the second part of the study, the participants were briefed on the study objectives. Their voluntary participation and confidentiality of their information were also ensured.

## Results

In the Classic Delphi stage, 76.3% of the experts were male and 23.7% female. The participants were also 28–52 years old and had a mean age of 34.7 ± 6.3 years. They also had a mean work experience of 9.9 ± 5.3 years and a mean HIS work experience of 6.6 ± 1.7 years, whereas 63.2% had a bachelor’s degree.

In the first round of the Classical Delphi Technique, the experts approved all the requirements associated with communication service, system architecture, security service and system response time. A requirement associated with system architecture, which did not receive the minimum score in the first round, was approved after being reassessed along with the newly-proposed requirements in the second round of the Classical Delphi Technique. The 73-item list of HIS technical requirements was ultimately confirmed with a mean score of 3.6 in the four domains. These requirements were met in 68.9% of the HISs run in the study hospitals. Tables 1, 2, 3, 4 and 5 present the results of the Delphi technique and evaluation of HISs technical requirements.

**Table 1:** The mean score of the technical requirements and the HIS evaluation results

Domains		Delphi	Evaluation			
		Mean Score	Yes	No	Total	Status
1	Communication service	3.49	102 (63.8)	58 (36.2)	160 (100)	Good
2	System architecture	3.53	283 (65.5)	149 (34.5)	432 (100)	Good
3	Security service	3.5	359 (72.4)	137 (27.6)	496 (100)	Good
4	System response time	3.82	61 (76.3)	19 (23.8)	80 (100)	Good
	Total	3.6	805 (68.9)	363 (31.1)	1168 (100)	Good

**Table 2:** The mean score of communication service requirements and the HIS evaluation results

	Item	Delphi	Evaluation	
		Mean Score	Yes	No
1	Electronically transferring data among different hospital departments	3.78	14 (87.5)	2 (12.5)
2	Exchanging data with other software systems	3.73	12 (75)	4 (25)
3	Transferring data among different software versions	3.71	12 (75)	4 (25)
4	Using standard protocols approved by the country’s competent authorities to exchange patient records and financial information	3.6	8 (50)	8 (50)
5	Simultaneous review of a file by multiple users	3.5	15 (93.8)	1 (6.2)
6	Having access to the data of other components from other locations based on the access level	3.5	16 (100)	–
7	Recording and modifying orders in different parts of the hospital and accessing these stations based on security and level of access	3.34	14 (87.5)	2 (12.5)
8	Calling the required developed services	3.28	2 (12.5)	14 (87.5)
9	Supporting communication with software through fax, WORD, spreadsheet, e-mail and the Internet	3.26	6 (37.5)	10 (62.5)
10	Consulting and communicating with physicians and specialists outside the hospital (audio-visual communication)	3.18	3 (18.8)	13 (81.2)
**Total**		3.49 ± 0.38	102 (63.8)	58 (36.2)

**Table 3:** The mean score of system architecture requirements and the HIS evaluation results

	Item	Delphi	Evaluation	
		Mean Score	Yes	No
1	Using standard databases	3.86	16 (100)	–
2	Handling the standard Persian language	3.84	16 (100)	–
3	Handling an unlimited number of clients	3.78	14 (87.5)	2 (12.5)
4	Availability of standard templates for output and input information	3.76	13 (81.2)	3 (18.8)
5	Installing the client and server easily and standard form.	3.73	16 (100)	–
6	Upgrading through the server easily and automatically	3.73	14 (87.5)	2 (12.5)
7	Employing standard programming languages	3.71	15 (93.8)	1 (6.2)
8	Compatibility with the international standards for client-server operating systems	3.71	11 (68.8)	5 (31.2)
9	Exporting data to different types of statistical programs	3.71	11 (68.8)	5 (31.2)
10	Generating customized reports	3.68	9 (56.2)	7 (43.8)
11	Application of server’s date and time rather than client’s date and time in the software	3.63	13 (81.2)	3 (18.8)
12	Execution of routines as services instead of manual execution	3.55	12 (75)	4 (25)
13	Being run in a network-connected mode using the client-server method	3.52	13 (81.2)	3 (18.8)
14	Providing predicted and routine reports	3.52	14 (87.5)	2 (12.5)
15	Adopting appropriate solutions to server connection among different units	3.5	11 (68.8)	5 (31.2)
16	Application of external devices and other devices in the system	3.5	15 (93.8)	1 (6.2)
17	Providing functional independence for clients of certain operating systems and platforms	3.47	7 (43.8)	9 (56.2)
18	Application of supported standards	3.47	8 (50)	8 (50)
19	Reporting through the Web Service	3.44	7 (43.8)	9 (56.2)
20	Providing all the technical specifications, relationships among tables, ERD, routines and among software classes as UML as well as other technical features of the database in writing and based on the RUP methodology for large projects or XP for small projects	3.44	4 (25)	12 (75)
21	Recording all the modifiable data and procedures in the database and avoiding storing them in the program code	3.39	9 (56.2)	7 (43.8)
22	Availability of a multilayer enterprise architecture for the design	3.34	6 (37.5)	10 (62.5)
23	Compatibility with the Web	3.28	6 (37.5)	10 (62.5)
24	Employing the procedures by using Commit and Rollback	3.28	5 (31.2)	11 (68.8)
25	Recording and editing data through the Web	3.23	4 (25)	12 (75)
26	Using open-source tools in the system design and production	3.07	6 (37.5)	10 (62.5)
27	Visibility of database contents and non-coding information	3.02	8 (50)	8 (50)
**Total**		3.53 ± 0.37	283 (65.5)	149 (34.5)

**Table 4:** The mean score of security service requirements and the HIS evaluation results

	Item	Delphi	Evaluation	
		Mean Score	Yes	No
1	Automatic and periodic backup options	3.89	15 (93.8)	1 (6.2)
2	Observing all the protection and security issues when accessing the database on the network	3.86	15 (93.8)	1 (6.2)
3	Providing user identity by placing username and password based on the user access level	3.84	16 (100)	–
4	Defining the access level based on layering data to preserve valuable information	3.78	16 (100)	–
5	Security in web applications	3.73	6 (37.5)	10 (62.5)
6	Logging user performance and reporting it to the system administrator, log management	3.71	12 (75)	4 (25)
7	Automatic retrieval of information whenever necessary	3.71	14 (87.5)	2 (12.5)
8	Equipping servers and clients with the antivirus employed by users	3.68	15 (93.8)	1 (6.2)
9	Providing a program for electronically storing and archiving information at specific intervals	3.68	10 (62.5)	6 (37.5)
10	Not displaying encryption as text	3.65	16 (100)	–
11	Supporting a standard locking mechanism to prevent updates by unauthorized individuals	3.63	9 (56.2)	7 (43.8)
12	Setting the password as text/number	3.6	12 (75)	4 (25)
13	Forming a personal information file including user characteristics required for determining the security service level	3.6	13 (81.2)	3 (18.8)
14	Defining functional roles and relationships with access levels	3.6	14 (87.5)	2 (12.5)
15	Recording and reporting all logins and logouts from the software and accessing all the appropriate features for registration such as username, workstation IP and MAC	3.6	12 (75)	4 (25)
16	Manual retrieval of information whenever necessary	3.55	15 (93.8)	1 (6.2)
17	Defining sections of the specific and confidential information	3.55	11 (68.8)	5 (31.2)
18	Resetting a password used	3.5	15 (93.8)	1 (6.2)
19	Application functionality in workstations under domain	3.47	14 (87.5)	2 (12.5)
20	Lack of access to the database except for the interface	3.42	11 (68.8)	5 (31.2)
21	Remote monitoring and control technology	3.36	6 (37.5)	10 (62.5)
22	Compatibility with hardware firewalls	3.34	12 (75)	4 (25)
23	Restricting user access to other operating system resources	3.28	13 (81.2)	3 (18.8)
24	Manual backup options	3.28	16 (100)	–
25	Supporting digital signatures	3.23	2 (12.5)	14 (87.5)
26	Lack of a random port use	3.23	5 (31.2)	11 (68.8)
27	Not requiring local administrators	3.21	13 (81.2)	3 (18.8)
28	Authentication via domain	3.18	11 (68.8)	5 (31.2)
29	Providing access to the system using different IPs and routing capabilities	3.15	6 (37.5)	10 (62.5)
30	Using name (as defined in DNS) and not depending on IP and computer name	3.15	9 (56.2)	7 (43.8)
31	Supporting the biosensor technology for logon	3.02	5 (31.2)	11 (68.8)
**Total**		3.5 ± 0.39	359 (72.4)	137 (27.6)

**Table 5:** The mean score of system response time requirements and the HIS evaluation results

	Item	Delphi	Evaluation	
		Mean Score	Yes	No
1	Fast search in sections with massive amounts of information	3.86	11 (68.8)	5 (31.2)
2	Easily and quickly reporting	3.84	13 (81.2)	3 (18.8)
3	Responding to user requests for specific operations within an acceptable response time	3.81	9 (56.2)	7 (43.8)
4	Providing uninterrupted access to the system 24 h a day	3.81	16 (100)	–
5	Acceptable processing time	3.78	12 (75)	4 (25)
**Total**		3.82 ± 0.29	61 (76.2)	19 (23.8)

Table 2 presents communication service domain as 10 items; showing a mean score of 3.49 ± 0.38 using the Delphi method and suggesting that the study HISs met 63.8% of the requirements of communication service domain.

According to Table 3, the 27-item system architecture domain with a mean score of 3.53 ± 0.37 was approved, which has been met in 65.5% of implemented HISs.

According to Table 4, the 31-item service security domain with a mean score of 3.5 ± 0.39 was approved, which observed in 72.4% of the implemented HISs.

According to Table 5, the 5-item system response time domain with a mean score of 3.82 ± 0.29 was confirmed, which observed in 76.2% of the implemented HISs.

## Discussion

Experts approved HIS technical requirements in four domains, i.e. communication service (10 items), system architecture (27 items), security service (31 items) and system response time (5 items). Meeting the requirements in the study hospitals was found to be at good levels (68.9%).

Technical requirements for the HIS are largely addressed in literature using a researcher-made checklist, including a very limited number of items (4–7 items), on minor technical requirements [14, 17]. Even the requirements defined by the Iranian Ministry of Health and Medical Education in two domains of security services and communication services and 23 items [21] are not comparable with the number of items and domains defined in the present study. The present study designed data collection tools in a way that more specific and comprehensive domains and items were provided for evaluating the HIS on a national scale; nevertheless, technological advances require that further studies be conducted on diverse requirements and domains in the future.

The experts confirmed communication service as a domain of the technical requirements. The requirements for the study HISs associated with communication service were met in 63.8% of the cases, which was considered good though lower than the compliance rates of the other domains. Moreover, the standard protocol approved by the national healthcare authorities for patient information exchange was met in only half of the information systems of the research population. The National Coordination Office of Health Information Technology of America emphasizes the need for employing and observing standards and credible settings for interoperability and sharing and using electronic health information given the fact that these standards can prove useful in designing modern systems [26, 27]. Investigating the implementation challenges of the HIS found the poor integration of different systems to be a potential source of problems [28]. In Hungary, Aggod-Feko found HISs capable of communicating within hospitals, although they reported limited potential for inter-communication with outside the hospital [29]. Investigating the acceptability of the health information technology in seven industrial countries suggested that, despite their popularity, EHRs were yet lagging behind the health information exchange [23]. Numerous studies also reported very low levels of interoperability among HISs as low scores in assessments [30–33]. The lack of interoperability among HISs increases paperwork [34] and restricts healthcare organizations’ use of the product portfolio of a software supplier in a way that their data can be stored in the specific format of the supplier and cannot be easily transferred to other systems [35]. According to users, working with integrated systems could reduce working hours and increase work speed [6]. A high-performance information exchange system can indirectly contribute to nursing care [36] and reduce patient re-admission rates [37]. The HIS failure in terms of information exchange imposes financial burdens on hospitals and causes failure to refund the costs to healthcare organizations [38]. Exchanging information among HISs also helps reduce the costs of clinical communication among healthcare providers and transmission of laboratory and imaging reports [39]. Investigating Electronic Medical Records (EMRs) by Walker et al. showed that interoperability and health information exchange can result in an annual saving of $77 million [40]. A unified and centralized EHR approach for individual patients requires the provision of regional Electronic Patient Records (EPRs) with some degree of internal exchange [41], although the integration of HISs into other systems is known as the main feature of the fourth and current HIS generation [6]. A special attention should be paid to proposing a framework of communication service requirements for the HIS that includes information exchange standards at the system design phase given the following points: a) the key role of information exchange in information systems, b) the need for developing interoperable systems, c) prevention of organizational dependence on the products of a particular company, d) the effect of a lack of data integrity on treatment and costs and e) inhibiting the development and implementation of EHRs by heterogeneous software forests.

The system architecture was an important domain of the technical requirements. Compliance with the system architecture requirements in the study HISs were found to be proper (65.5%), which were yet lower than that of two other domains. In software development, system architecture determines the system development model and tools and environment development [42]. Multiple distributed systems developed in different programming languages demand more mechanisms in terms of practical communication [43]. Moreover, service-oriented architectures can be used to define IT infrastructures that enable different programs to exchange data and cooperate in the business process regardless of the type of the operating system and programming language through which the program is designed. Moreover, diverse healthcare programs can be run on the web based on messaging standards to exchange office and clinical data [44]. Systems designed based on diverse types of platforms and programming languages appear to make it difficult to develop HIS projects by creating numerous heterogeneous systems in an organization. Employing open-source tools, web-based implementation and supported standards as part of the framework of system architecture requirements can therefore assist with future software development.

Evaluating the information systems of the study population showed good levels (72.4%) of meeting security service as a domain of the technical requirements. Security issues hinder the application and implementation of information systems in the healthcare sector [45]. Security concerns associated with EHRs have also been reflected in literature [46–48]. Moreover, levels of technical security were reported as high in some health centers and moderate in certain hospitals [49]. In yet other studies, over 50% of respondents reported the security of their health data at good/moderate levels [32, 50]. Research suggests neither a specific policy for patient access to information nor a punishment for unauthorized access to information. Users can access the system using the same password with no time limitation given that no expiration dates are usually defined for the passwords [49]. The system must incorporate security mechanisms for controlling user access in terms of tasks or classified access [51]. An effective access control system ensures that only authorized users can access system resources [4]. On the other hand, the HIS should provide easy access to healthcare information at healthcare institutions [52]. The user-friendly techniques commonly used to guarantee data confidentiality and user validation include setting passwords, biometric techniques, smart cards and certifications [53]. The access control mechanisms attempt to prevent illegal operations before they occur [54]. According to the majority of IT experts, making a backup should be given top priority over the other security requirements of nursing data in EHRs [55]. Providing encrypted incremental backups for information was reported as a security measure in EHRs protection [56]. Given the effects of the HIS used for the security management of patient data on the care quality, the rights of patients and healthcare professionals and their working practices [57], patients may withdraw their information owing to their distrust of the HIS security, which is health-threatening [58]. Solving the security and privacy problems of the HIS is therefore crucial for meeting potential data protection challenges [59]. Hospital authorities should follow a standardized set of instructions to enhance information security [60, 61]. Different researchers have reported different security levels. Given the key role of data in providing patient care, data backups and data access control are known as the major security concerns of the systems. The implemented HIS should therefore incorporate a security framework that guarantees system security and facilitates access to information for authorized individuals.

The HISs evaluation showed good levels (76.3%) of meeting system response time as a domain of HIS ​​technical requirements approved by the experts. System response time is a major technical challenge of the HIS [28, 62]. The dissatisfaction with this domain highlighted in numerous studies was reported as an obstacle to applying EHRs and the main reason for their abandonment by users [62–64]. Organizations that heavily depend on computerized systems to deliver patient care require downtime as low as 0% and business continuity procedures to ensure safety and patient care continuity [65]. Timely high-speed system access identified as an important efficiency factor is therefore a general technical dimension of the HIS [51, 52]. A low system response time reduces the likelihood of the acceptance of the system by users and its successful implementation [66]. To enhance the system efficiency and consequently user satisfaction, the importance of the requirements for system response time is recommended to be emphasized when selecting the system.

## Conclusion

A 73-item framework was designed to address all the HIS technical requirements in four domains, i.e. communication service, system architecture, security service and system response time. The possibility of defining and adding other items to this framework to meet the requirements for individual HISs makes it applicable to different settings. It can therefore benefit other countries depending on their degree of development and progress in implementing the HIS. These requirements can be used in the lifecycle of information systems to help customers select a system that meets minimum user requirements for the HIS. Determining these requirements can also assist the designers and developers of HISs in adapting the software program to user needs. Evaluating the HIS in the study population suggested good scores in meeting HIS technical requirements, although communication service and system architecture respectively received the lowest scores. Constantly developing heterogeneous HISs in hospitals makes the integration of these systems difficult to achieve given their different designs and their implementation according to different principles. The difficult transfer of the patient data stored in these systems to other systems is also a major challenge and obstacle to establishing interoperability among different systems in terms of developing EHRs and modifying the systems according to the individual needs of organizations and users. Sending the results of evaluating the HIS as feedback to software providers can assist them in improving their programs and meeting technical requirements.

## Supplementary information

**Additional file 1.** Technical requirements questionnaire.

## Data Availability

The datasets used and/or analyzed in the present study can be made available by the corresponding author upon official requests.

## Abbreviations

DNS: Domain Name System
EHRs: Electronic Health Records
EPRs: Electronic Patient Records
EMRs: Electronic Medical Records
ERD: Entity Relationship Diagram
FGD: Focus Group Discussion
HIS: Hospital Information System
IT: Information Technology
IP: Internet Protocol
MAC: Machine Access Control
RFP: Request for Proposal
RUP: Rational Unified Process
UML: Unified Modeling Language
