# Supplementary material for: Technical requirements framework of hospital information systems: design and evaluation
Source: BMC Med Inform Decis Mak. 2020 Apr 3;20:61. doi: 10.1186/s12911-020-1076-5 (PMC7119017; doi:10.1186/s12911-020-1076-5)
Supplement: Supplementary file 1 — Additional file 1. Technical requirements questionnaire. [file 12911_2020_1076_MOESM1_ESM.docx]

**Final Questionnaire Sample**

Designing a framework for technical HIS requirements in Iran (The first and second stages of Delphi technique)

Dear participant

The present questionnaire was designed in order to conduct a study entitled “Designing a framework for technical HIS requirements in Iran”. The study may not reach its purpose unless you devote time and precision to answering questions provided. Thank you for your kind cooperation.

The questionnaire includes two sections of general and technical questions. There are no names on the forms and all the information provided will be kept private.

Section 1 (General Questions)

Sex: Male…… Female……

Age: ……

Education: Bachelor…… Masters…… Ph.D……

Field of study:……

Employment status: Official…… Periodical…… Contract……

Organization type:……….. Organization name:……….

Years of work experience:………. Software provider name:……….

Period of HIS launch in your work environment:……….

Period of work experience with the HIS:……….

Section 2 (Technical Questions)

Please provide us with your opinion regarding the inclusion of any of the following items in the framework of technical HIS requirements. In case any further items are needed to be added to the questionnaire, write it on the form.

Technical Requirements

| Communication Services | | Strongly Agree | | | Agree | No Idea | | Disagree | | | Strongly Disagree | | |
| --- | --- | --- | --- | --- | --- | --- | --- | --- | --- | --- | --- | --- | --- |
| 1 | Ability to transfer data electronically among different hospital departments |  | | |  |  | |  | | |  | | |
| 2 | Ability to exchange data with other software systems |  | | |  |  | |  | | |  | | |
| 3 | Ability to transfer data among different software versions |  | | |  |  | |  | | |  | | |
| 4 | Ability to use the standard protocols approved by the country's competent authorities to exchange patient records and financial information |  | | |  |  | |  | | |  | | |
| 5 | Ability to simultaneously review a file by multiple users |  | | |  |  | |  | | |  | | |
| 6 | Ability to access data of other components from other locations based on the access level |  | | |  |  | |  | | |  | | |
| 7 | Ability to record and modify orders in different parts of the hospital and accessing these stations based on security and level of access |  | | |  |  | |  | | |  | | |
| 8 | Ability to call the required developed services |  | | |  |  | |  | | |  | | |
| 9 | Ability to support communication with software through fax, WORD, spreadsheet, e-mail and the Internet |  | | |  |  | |  | | |  | | |
| 10 | Ability to consult and communicate with physicians and specialists outside the hospital (audio-visual communication) |  | | |  |  | |  | | |  | | |
| Other: | | | | | | | | | | | | | |
| System Architecture | | | Strongly Agree | | Agree | | No Idea | | | Disagree | | | Strongly Disagree |
| 1 | Ability to use the standard databases | |  | |  | |  | | |  | | |  |
| 2 | Ability to handle the standard Persian language | |  |  | |  | | |  | | |  | |
| 3 | Ability to handle an unlimited number of clients | |  |  | |  | | |  | | |  | |
| 4 | Ability to available of standard templates for output and input information | |  |  | |  | | |  | | |  | |
| 5 | Ability to install the client and server easily and standard form | |  |  | |  | | |  | | |  | |
| 6 | Ability to upgrade through the server easily and automatically | |  |  | |  | | |  | | |  | |
| 7 | Ability to use the standard programming languages | |  |  | |  | | |  | | |  | |
| 8 | Ability to comply with the international standards for client-server operating systems | |  |  | |  | | |  | | |  | |
| 9 | Ability to export data to different types of statistical programs | |  |  | |  | | |  | | |  | |
| 10 | Ability to generate custom reports | |  |  | |  | | |  | | |  | |
| 11 | Ability to use server's date and time rather than client's date and time in the software | |  |  | |  | | |  | | |  | |
| 12 | Ability to execute routines as services instead of manual execution | |  |  | |  | | |  | | |  | |
| 13 | Ability to run in a network-connected mode using the client-server method | |  |  | |  | | |  | | |  | |
| 14 | Ability to have predicted and routine reports | |  |  | |  | | |  | | |  | |
| 15 | Ability to adopt an appropriate solution to server connection among different units | |  |  | |  | | |  | | |  | |
| 16 | Ability to use the external devices and other devices in the system | |  |  | |  | | |  | | |  | |
| 17 | Ability to give independence to clients of specific operating systems and platforms | |  |  | |  | | |  | | |  | |
| 18 | Ability to use supported standards | |  |  | |  | | |  | | |  | |
| 19 | Ability to report through the Web Service | |  |  | |  | | |  | | |  | |
| 20 | Ability to provide all technical specifications, relationships among tables, ERD, routines and among software classes as UML as well as other technical features of the database in writing and based on the RUP methodology for large projects or XP for small projects | |  |  | |  | | |  | | |  | |
| 21 | Ability to record all the modifiable data and procedures in the database and avoid storing them in the program code | |  |  | |  | | |  | | |  | |
| 22 | Ability to available on multilayer enterprise architecture in software design | |  |  | |  | | |  | | |  | |
| 23 | Ability to run on the Web | |  |  | |  | | |  | | |  | |
| 24 | Ability to use the Commit and Roll Back Standards in using routines | |  |  | |  | | |  | | |  | |
| 25 | Ability to record and edit data through the Web | |  |  | |  | | |  | | |  | |
| 26 | Ability to use the open-source tools in the system design and production | |  |  | |  | | |  | | |  | |
| 27 | Ability to view database contents and non-coding information | |  |  | |  | | |  | | |  | |
| Other: | | | | | | | | | | | | | |
| Security Service | | | Strongly Agree | Agree | | No Idea | | Disagree | | | | Strongly Disagree | |
| 1 | Ability to back up periodically and automatically | |  |  | |  | |  | | | |  | |
| 2 | Ability to observe all the protection and security issues when accessing the database on the network | |  |  | |  | |  | | | |  | |
| 3 | Ability to provide user identity by placing username and password based on the user access level | |  |  | |  | |  | | | |  | |
| 4 | Ability to define the access level based on layering data to preserve valuable information | |  |  | |  | |  | | | |  | |
| 5 | Ability to secure web applications | |  |  | |  | |  | | | |  | |
| 6 | Ability to log the user's performance and reporting it to the system administrator, log management | |  |  | |  | |  | | | |  | |
| 7 | Ability to retrieve information automatically whenever necessary | |  |  | |  | |  | | | |  | |
| 8 | Ability to equip servers and clients with the antivirus employed by users | |  |  | |  | |  | | | |  | |
| 9 | Ability to provide a program for electronically storing and archiving information at specific intervals | |  |  | |  | |  | | | |  | |
| 10 | Ability to not display the encryption as a text | |  |  | |  | |  | | | |  | |
| 11 | Ability to support the standard locking mechanism to prevent updates by unauthorized individuals | |  |  | |  | |  | | | |  | |
| 12 | Ability to set the password as text/number | |  |  | |  | |  | | | |  | |
| 13 | Ability to form a personal information file including user characteristics required for determining the security service level | |  |  | |  | |  | | | |  | |
| 14 | Ability to define functional roles and relationships with access levels | |  |  | |  | |  | | | |  | |
| 15 | Ability to record and report all logins and logouts from the software and access to all the appropriate features for registration such as username, workstation (IP) and MAC | |  |  | |  | |  | | | |  | |
| 16 | Ability to retrieve information Manually whenever necessary | |  |  | |  | |  | | | |  | |
| 17 | Ability to define sections of the specific and confidential information | |  |  | |  | |  | | | |  | |
| 18 | Ability to reset the password | |  |  | |  | |  | | | |  | |
| 19 | Ability to software functionality in workstations under domain | |  |  | |  | |  | | | |  | |
| 20 | Ability to access the database except for the interface | |  |  | |  | |  | | | |  | |
| 21 | Ability to remote monitoring and control technology | |  |  | |  | |  | | | |  | |
| 22 | Ability to adoption with hardware firewalls | |  |  | |  | |  | | | |  | |
| 23 | Ability to restrict user access to other operating system resources | |  |  | |  | |  | | | |  | |
| 24 | Ability to back up manually | |  |  | |  | |  | | | |  | |
| 25 | Ability to support digital signature | |  |  | |  | |  | | | |  | |
| 26 | Ability to not use the random port | |  |  | |  | |  | | | |  | |
| 27 | Ability to no need to local administrators | |  |  | |  | |  | | | |  | |
| 28 | Ability to authentication via domain | |  |  | |  | |  | | | |  | |
| 29 | Ability to access the system using different IPs and routing capabilities | |  |  | |  | |  | | | |  | |
| 30 | Ability to use name (as defined in DNS) and not depending on IP and computer name | |  |  | |  | |  | | | |  | |
| 31 | Ability to support the biosensor technology for logon | |  |  | |  | |  | | | |  | |
| Other: | | | | | | | | | | | | | |
| System Response Time | | Strongly Agree | | | Agree | | No Idea | | | Disagree | | | Strongly Disagree |
| 1 | Ability to fast search in sections with massive amounts of information |  | | |  | |  | | |  | | |  |
| 2 | Ability to report easily and quickly |  | | |  | |  | | |  | | |  |
| 3 | Ability to respond within acceptable system response time to user requests for specific operations |  | | |  | |  | | |  | | |  |
| 4 | Ability to access the system 24 hours a day without any interruption |  | | |  | |  | | |  | | |  |
| 5 | Ability to performance within acceptable processing time |  | | |  | |  | | |  | | |  |
| Other: | | | | | | | | | | | | | |
